# Fulton County Medical Society, Regular Meeting May 7, 1914

**Published:** 1914-05

**Authors:** R. R. Daly


### FULTON COUNTY MEDICAL SOCIETY
### REGULAR MEETING MAY 7, 1914.

Reported by R. R. Daly M. D.

Dr. L. Sage Hardin exhibited a case of Goitre in a child. She had had typhoid fever, pyelitis and adenectomy in her career, but continued peevish and in ill health. Nutrition was interfered with without apparent reason until the goitre made itself manifest.

Lately it has grown until it will change size and the character of the voice in one conversation. He expects operation at a suitable time.

J. R. McCord read a paper on "The Present Status of Pituitrin in Obstetrics." It appears elsewhere in the Journal.

Dr. J. L. Campbell said that in an experience of twenty-five cases, his conclusions were that pituitrin is of value in stopping post partum haemorrhage and in the bleeding from abortion. If given at all in labor, one should wait until the head is engaged. When used in a case of face presentation, it did no good and the child was delivered with forceps. There was profuse post partum hemorrhage ensuing. One should be particularly careful as to high blood pressure when this considering drug. It is contraindicated at this time.

Dr. John Denton said that there were three extracts made from the gland and that pituitrin was the one Edgar did not advise. Denton has used it successfully in three cases of dry labor. It had prevented or avoided the use of forceps. In another case where the placenta was adherent, four doses have caused inversion of the uterus. Menorrhagia at the menopause is helped by it more than by curettage. However, it tends to prevent cathetrization in post operative cases.

Dr. E. G. Jones gave Lantern Demonstrations of Goitre.

These showed the pathology of the gland in the different forms of degeneration that affect it, and on this basis the treatment was outlined.

Dr. E. C. Davis said that the exhibit was an index of the painstaking investigation that is being done with great advantage here in Atlanta. Goitre is one of the great subjects of the day and operations in properly investigated and selected cases are very satisfactory. The studies showed what was needed and pointed out the necessity for preparation of the cases. The period of rest in bed for several days with cold applications to the neck was essential to success in operation.

Dr. L. Sage Hardin said he was deeply interested in the differentiation of types as shown by Jones. The acinous type particularly attracted his attention. The occurrence of the disease varied as to locality, age of patient and the presence of other diseases in his experience. So far as the valleys and lack of sunlight are concerned, we all know the history of cretinism without exactly knowing what it means. He has noted in addition that attacks come on at certain sexual crises, puberty, beginning of childbearing, as in early married life, etc. Malignancy follow the cystic type as a rule. Elsner reports 500 cases in connection with uterine fibroid. As to sex, the disease occurs more in females; the proportion being 9 to 1. As to preparation for operation in addition to rest in bed and cold applications, he uses the alkaline treatment of soda bicard per rectum. This reduces the pulse rate and pressure.

At this time Dr. Hardin exhibited another case that was late in coming but had special relevance. A young boy just approaching puberty has been recently suffering with acute nephritis with albumen and casts. He had also shown some signs of chorea. These subsided under rest treatment and then the goitre which was the prime cause of all the symptoms appeared. It is still a medical case and he hopes it will recover without surgical intervention.

Dr. Jones in closing said, that any and all depressing states made an increased demand upon the thyroid. The thyroglobulin is a tonic and assists in this way to carry the burden.

But at the same time there is secreted an excess of the nucleo proteid product that causes the symptom complex of hyperthyroidism. Any Goitre long present will cause serious trouble.

---

### REGULAR MEETING MAY 21, 1914.

MEETING MAY 21, 1914.

Dr. John F. Denton exhibited a specimen of full term tubal pregnancy, removed without rupture after having been in the abdomen for two years. The woman was operated on for fibroid and it was not until the mass was removed and opened that its true nature was discovered. The foetus occupies the same position in the tube that it would have in the uterus. There was no absorption of tissue except that the amniotic fluid was gone. There never had been any toxic symptoms. The woman told of having felt two years before, but the swelling came down and she concluded she had been mistaken as to pregnancy. She is a negro and had been in the hands of negro doctors who had told her various things about it.

Dr. L. Amster read a paper "A Few Remarks on the Diseases of the Oesophagus, Cardiospasm, and Gastroscopy."

He exhibited several modern instruments for the determination of the location of strictures and ulcers, as well as for the treatment of the diseases indicated.

Dr. S. R. Roberts said these were new instruments to him and that he welcomed them to the community together with the skill necessary to manipulate them. Men in general work often need the assistance of a special operator in this line, particularly in nervous cases. The Kallman Sound takes the place of the X-ray in finding abrasions that may become ulcers and cause strictures. As to surgery, Janeway of New York has developed results of surgical work in the oesophagus that are excellent and he prophesies that there will be further benefits accruing.

Dr. W. M. Dunn read a paper on "Surgical Treatment of Burns."

It will appear together with the discussion in a later number of the Journal.

Dr. W. S. Kendrick gave a comprehensive talk on "Life Insurance Examination, Responsibilities, etc."

Dr. E. L. Griffin asked as to whether the statistics of any cases showed mortality decrease or longevity increase. Dr. Kendrick said he did not know.

Dr. M. T. Davis said that the medical inspecitions of schools and factories tended to increase longevity and had its influence upon all statistics, but they could not be determined as yet.

Dr. Person asked as to the effect of syphilis in life risks.

Dr. R. R. Daly said that many companies having studied the actual tables have concluded that they would be safe in issuing group insurance to certain kinds of occupations without any examination and that this was being done. It did not speak very well for the care taken in the medical examinations as a whole. Bad risks should be eliminated by the careful examiner and his mortality should show this.

One matter came frequently to his attention. Applicants demurred against removing their clothing for complete examination of the thorax and said that this or that eminent man had previously examined them without this requirement. He could not pretend to determine a heart murmur unless he could get close enough to hear it and he was not wizard enough to do this through a starched shirt or with any other obstruction to hearing. He had tried but he couldn't do it, and he was unable to understand how anyone could. As to syphilis, one large company had informed him that the combined actual experience showed that a man having had syphilis and recovered from it was a poorer risk the longer he lived. That is, he was less likely to live his expectancy 10 or 15 years after his disease than he was 2 or 3 years after it. Most of the large companies decline to take any syphilitic cases. The importance of alcohol is stressed in the front page of every examination blank and the companies are coming more and more to limit their risks where alcohol is indulged in either periodically or regularly. The importance of Blood Pressure is coming to the fore, and though there has not been time for many actual deductions, the companies he is familiar with are calling for steadily increasing investigations in this matter.

Dr. W. H. Hancock spoke against making examinations outside of one's office. The conditions were never such as to give freedom to examination and often the led to deception as to urine specimens and the like. One could not question accurately as to history of disease when others were around as they usually were when he doctor could not control the situation as he does in his office. Another matter was the deceit of passing negroes. This has been done by unscrupulus men to the detriment of the reputation of the entire profession.

Dr. J. C. White spoke of the temptations that come to the country doctor to pass men when he knows everyone in the community and they all know and rely upon him to do almost everything for them. The agent presses him and the applicant gets furious if not passed. The doctor loses business in bad cases and there is no end of trials for him. It is small wonder that in cases where the question is not too great he gives the applicant the benefit of the doubt.

Dr. Kendrick said in closing that his company paid all cases of honest examination whether the risk was passed or not and he supposed all companies did the same. He would not pass any case of albumen urea no matter if every indication was that it was transient. He would wait. As to syphilis, he took no risks. As to alcohol, the steady drinker was the one he feared and would reject. The man who went on an occasional spree was not the highest class of risk, of course, but there was little danger to his arteries from this cause.

Dr. S. R. Roberts related a case of the use of Salvarsan where there was cyanosis and grave danger. He believes we have come to a time when there must be vastly more caution in the use of this drug and he calls attention to Ehrlic's teaching of beginning low and ending high as to the dosage. Begin with very small amounts and see what will happen before going to the higher amounts. A preparation of the patient with mercury is most advantageous according to the same high authority. Incidentally but of great importance is the knowledge that Adrenalin will counteract the syncope in these cases.

The syncope is a sudden and profound dilatation of the blood vessels of the brain and adrenalin, as Dr. Gaines of Atlanta has pointed out many months ago, will cure this trouble.

